# LigandRFs: random forest ensemble to identify ligand-binding residues from sequence information alone

**DOI:** 10.1186/1471-2105-15-S15-S4

**Published:** 2014-12-03

**Authors:** Peng Chen, Jianhua Z Huang, Xin Gao

**Affiliations:** 1 Computer, Electrical and Mathematical Sciences and Engineering Division, King Abdullah University of Science and Technology (KAUST), Thuwal 23955-6900, Saudi Arabia; 2 Institute of Health Sciences, Anhui University, Hefei, Anhui 230601, China; 3 Department of Statistics, Texas A&M University, College Station, TX 77843-3143, USA

## Abstract

**Background:**

Protein-ligand binding is important for some proteins to perform their functions. Protein-ligand binding sites are the residues of proteins that physically bind to ligands. Despite of the recent advances in computational prediction for protein-ligand binding sites, the state-of-the-art methods search for similar, known structures of the query and predict the binding sites based on the solved structures. However, such structural information is not commonly available.

**Results:**

In this paper, we propose a sequence-based approach to identify protein-ligand binding residues. We propose a combination technique to reduce the effects of different sliding residue windows in the process of encoding input feature vectors. Moreover, due to the highly imbalanced samples between the ligand-binding sites and non ligand-binding sites, we construct several balanced data sets, for each of which a random forest (RF)-based classifier is trained. The ensemble of these RF classifiers forms a sequence-based protein-ligand binding site predictor.

**Conclusions:**

Experimental results on CASP9 and CASP8 data sets demonstrate that our method compares favorably with the state-of-the-art protein-ligand binding site prediction methods.

## Background

Protein-ligand binding is important for some proteins to perform their functions. Protein-ligand binding sites are the residues of proteins that physically bind to ligands. A ligand is a signal triggering molecule, binding to a site on a target protein. In biochemistry, a ligand is a substance, usually a small molecule, that forms a complex always with a molecule to serve a biological purpose. For instance, oxygen is poorly soluble in aqueous solutions and cannot be perfectly carried to tissues if it is only dissolved in blood serum. However, none of the amino acid side chains in proteins is suited for the reversible binding of oxygen molecules. The function is always fulfilled by certain transition metals having a strong tendency to bind oxygen, such as iron and copper. Most commonly iron is used for the oxygen transportation. Myoglobin (PDB: 3RGK) is an iron- and oxygen-binding protein to facilitate the oxygen diffusion in muscles. It is a single polypeptide consisted of 153 or 154 amino acid residues, which is found in almost all mammals, primarily in muscle tissue. Commonly, there are several ligand categories: "metal ions" (e.g., Ca, Zn, Fe, and Mg), "inorganic anions" (e.g., SO4 and PO4), "DNA/RNA" for poly-ribonucleic acid binding sites, and "organic ligands" for cofactors, substrates, and receptor agonists/antagonists (e.g., NAD, FAD, ATP, SAM, CoA, and PLP) [1], and so on.

A number of methods applied nuclear magnetic resonance (NMR) spectroscopy [2-9] or X-ray [10] to determining protein structures. Such structural information is essential to determine the ligand-binding sites. Pintacuda et al. employed lanthanide ions for the determination of protein-ligand binding sites [2]. Ziarek et al. emphasized a semi-automated throughput-focused method to identify practical aspects of binding site characterization and structure determination of protein-ligand complexes, by automated and semi-automated NMR assignment methods [4]. Since experimental efforts to determine ligand-binding sites are always time-consuming, computational methods are needed that can assist in the identification of such sites.

Most computational approaches searched for similar or homologous structures of the query sequence to determine its ligand-binding sites from the homologous structures [1,11-13]. For instance, in the CASP9 competition, all top performing groups were based on the structure-based approach. Although they yielded good predictions (the average Matthews correlation coefficient of 0.62 for the top 10 performing groups), such structure-based techniques are restricted by the limited number of available protein structures. Therefore, sequence-based approaches are particulary useful when similar structural information is not available. A number of sequence-based methods have been developed to predict ligand-binding states [14-16]. Passerini and co-workers introduced a method for identifying histidines (in either of two states: free or metal bound) and cysteines (in either of three states: free, metal bound, or in disulfide bridges) that participated in binding of several transition metals and iron complexes [15]. Shu et al. developed a method combining support vector machines (SVM) and homology-based predictions to predict zinc-binding sites (Cys, His, Asp and Glu) in proteins from their amino acid sequences [16]. Moreover, some sequence-based predictors attended the CASP9 competition [17].

However, the problem of whether ligand-binding sites can be predicted from sequence information remains open. Little progress has been made on the sequence-based ligand-binding site prediction. Kauffman and Karypis proposed a method that combined machine learning and homology information for the sequence-based ligand-binding site prediction [18]. However, the method did not perform well. In this paper, we propose a sequence-based approach, LigandRFs (Ligand binding site prediction by the ensemble of Random Forest classifiers), to identify protein-ligand binding residues based on the co-evolutionary context of amino acid residues. First, due to the imbalanced samples between ligand-binding sites and non-binding sites, several data sets are constructed. Each of them is composed of the binding site subset (positive subset) and part of the non-binding site subset (negative subsets). All the negative subsets are disjoint with each other. Then a random forest (RF) classifier is learned on each data set. Experiments on the CASP8 and CASP9 data sets show that the consensus of these RF classifiers can yield good prediction on ligand-binding sites.

## Results

**Figure 1:**
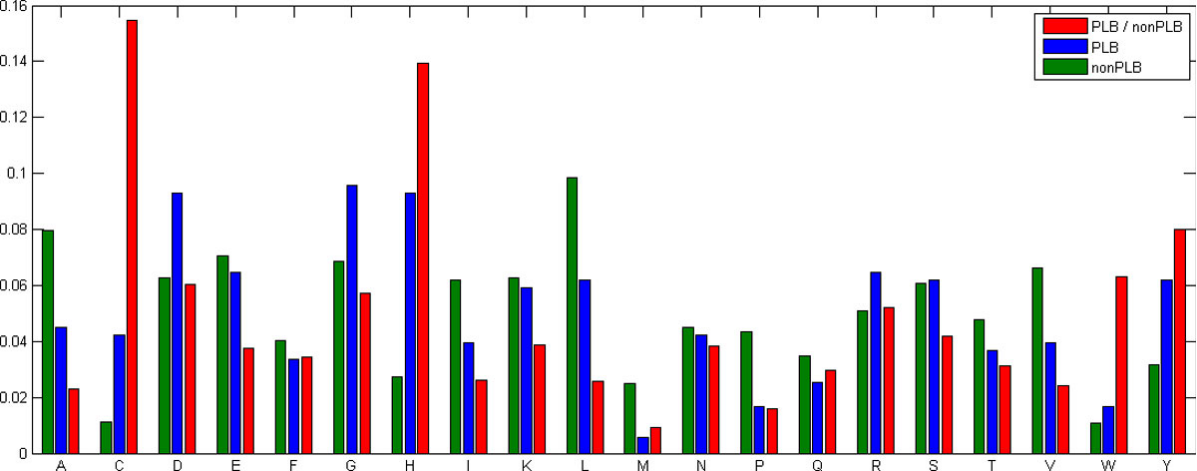
**Amino acid preferences for ligand binding sites (colored in blue) and non-ligand binding sites (colored in green)**. The corresponding ratio of the number of each amino acid in ligand binding to that in non-ligand binding is also illustrated here (in red). PLB and nonPLB in the figure legend denote protein-ligand binding and non-protein ligand binding, respectively.

We first analyzed the amino acid preferences for ligand binding sites and non-ligand binding sites. Figure 1 illustrates the preference comparison. It can be seen that amino acids, Asp, Gly and His, frequently occur in the ligand binding sites, while amino acids, Leu and Ala, are often regarded as non-ligand binding sites. However, it may not always be the case because our current data set is relatively small. Despite of that, Asp and His are considered as hydrophilic amino acids while Leu and Ala as hydrophobic ones in literature [19], which is partially in agreement with our statistics. In fact, hydrophilic amino acids seem to be more likely to be ligand binding sites.

### Prediction results on CASP9

In this work, we first used CASP8 data set to train our method and then test it on CASP9 protein ligand data set, both of the two data sets involve the same definition of protein ligand binding site. Also we use sliding window to encode the input vector for each residue of the protein, which is then inputted into a classifier to determine whether or not it is a ligand binding site. By using the sliding window with length seven, Table 1 shows the performance comparison of the 15 RF classifiers and that of the ensemble, on the prediction of all ligand binding sites that was extended to include the entire biologically relevant ligand. From Table 1 it can be seen that the ensemble of the 15 RF classifiers with majority voting performs well. It yields an MCC of 0.37 and an F1-score of 35.99%, which outperforms any individual RF classifier, where the best individual prediction, the 2*nd *classifier, achieves an MCC of 0.35 and an F1 of 33.59%.

**Table 1:** Overall prediction performance of the 15 RF classifiers and that of the ensemble with different votes on the CASP9 data set.

Individual					Ensemble				
**No**.	**Sen(%)**	**MCC**	**Prec(%)**	**F1(%)**	**No**.	**Sen(%)**	**MCC**	**Prec(%)**	**F1(%)**

1	57.15	0.30	25.25	29.83	1	87.86	0.19	9.98	16.85
2	59.40	*0.35*	31.13	33.59	2	84.48	0.22	12.58	19.95
3	63.24	0.31	24.35	29.38	3	81.86	0.24	14.05	21.49
4	65.76	0.33	25.20	31.14	4	80.28	0.25	15.39	22.67
5	44.50	0.32	34.65	31.24	5	78.84	0.27	16.65	24.09
6	57.83	0.31	26.22	30.47	6	75.99	0.27	17.38	24.58
7	59.12	0.33	29.19	31.62	7	74.81	0.28	18.86	26.00
8	59.23	0.32	27.18	31.16	8	73.32	0.29	20.21	27.03
9	67.88	0.30	22.80	22.56	9	72.63	0.30	21.17	28.04
10	51.21	0.31	28.62	31.21	10	71.21	0.31	23.42	29.34
11	46.99	0.31	30.96	30.53	11	69.51	0.32	24.69	30.19
12	64.50	0.30	23.61	28.96	12	67.31	0.33	25.72	30.64
13	61.25	0.31	25.28	29.20	13	64.93	0.33	26.68	31.07
14	40.51	0.31	38.38	30.41	14	62.01	0.34	28.78	32.46
15	59.98	0.31	26.08	30.50	15	56.96	*0.37*	34.97	35.99

The left part shows the performance of each individual classifier, and the right shows the performance of the ensemble of the 15 classifiers with different votes, i.e., the ensemble predicts a residue to be ligand binding site if a number of RF classifiers predict it to be a ligand binding site residue. Here the ensemble with majority vote predicts a residue to be ligand binding site if all of the 15 RF classifiers predict it to be a binding residue.

The italic number denotes the best performance by the measure of MCC.

Moreover, other sliding windows for the input encoding are conducted here. Table 2 shows prediction performance on different sliding windows and two ligand binding site groups: partial ligand sites including those only being in contact with atoms of the partial ligands, and all ligand sites including those being in contact with all atoms of the partial and the extended ligands. Among the different sliding windows in Table 2 window 7 performs the best in the case of the all ligand site group, while window 17 performs the best for the partial ligand site group. To reduce the effects of sliding window selection in encoding for input vectors, the combination technique (Eq. 1) is used and the performance is listed at the last row of the Table 2 respectively for the two binding site groups. It seems that the combination technique can reduce the effects of sliding window selection and achieves a little improvement.

**Table 2:** Prediction performance on different sliding windows for encoding input vectors on the CASP9 data set.

Window length	All ligand sites						Partial ligand sites					
	
	Sen (%)	Spe (%)	Acc (%)	MCC	Prec (%)	F1 (%)	Sen (%)	Spe (%)	Acc (%)	MCC	Prec (%)	F1 (%)
5	52.97	93.82	91.76	0.36	36.24	34.85	58.32	93.05	91.73	0.32	24.86	29.40
7	56.96	93.21	91.31	*0.37*	34.97	35.99	54.80	93.38	91.99	0.32	27.80	30.45
9	56.43	92.66	90.76	0.35	32.31	34.66	49.93	95.42	93.69	0.32	28.75	29.95
11	58.40	91.67	89.94	0.35	31.05	33.85	50.95	95.10	93.46	0.32	28.20	30.41
17	62.44	91.35	89.72	0.36	29.61	34.42	47.37	96.95	94.95	*0.34*	32.39	33.09
27	46.66	95.85	93.34	0.34	34.13	34.93	49.75	96.37	94.51	0.33	29.11	32.04
37	45.48	96.53	93.90	0.36	37.37	36.00	56.00	94.58	93.00	0.32	25.74	30.95
47	48.55	96.17	93.67	0.37	37.63	36.95	55.15	95.12	93.44	0.33	26.82	31.42
57	41.75	96.93	94.18	0.35	39.69	35.81	44.24	96.47	94.42	0.31	30.30	29.78

Combine	42.07	97.91	95.06	*0.40*	47.85	38.93	48.34	97.02	95.07	*0.34*	32.80	33.18

It contains prediction performance on the all ligand site group and the partial ligand site group. The ensemble of all sliding windows is shown at the last row of the table.

The italic number denotes the best performance on the measure of MCC.

We also output the prediction performance for each target in CASP9 and the details are shown in Table 3. The final prediction performance on CASP9 data set is obtained by the average of all the targets. Our predictor yields different performances over the data set, some targets obtaining good predictions and some ones performing worse. Statistics from Table 3 protein targets bound to metal ligands perform better than those bound to non-metal ions. Experiments showed that template-based prediction methods will perform much better than de novo methods in the context [1]. However, for targets T0604 and T0629, both of which are free modeling (FM) targets, our prediction on T0629 performs much better than that on T0604. The reason is seemingly that the target T0629 is bound to metal ligands while T0604 is bound to non-metal ligands. It should be noted that the ratio of the number of binding sites to the total number of residues of the target is not a significant factor on the prediction performance of our de novo method. It can be seen that the average ratios for metal binding sites and non-metal binding sites are 3.51% and 4.86%, respectively, but our predictor on those metal binding targets performs better than those non-metal ones, achieving an improvement of 0.23 by MCC.

**Table 3:** Performance and information on each of the CASP9 targets.

Target	Target PDB id	Number of residues	Chemical Class	Sen (%)	Prec (%)	MCC	Ratio (%)^§^
T0518	3NMB	288	Metal	57.14	57.14	0.56	2.43
T0521	3MSE	179		33.33	33.33	0.30	5.03
T0529	3MWT	569		50.00	9.09	0.20	0.70
T0539	2L0B	81		37.50	50.00	0.38	9.88
T0548	3NNQ	106		100.00	66.67	0.81	3.77
T0582	3O14	222		100.00	66.67	0.81	1.80
T0585	3NE8	234		60.00	75.00	0.66	2.14
T0625	3ORU	233		66.67	22.22	0.37	1.29
T0629	2XGF	216		100.00	73.68	0.85	6.48
T0635	3N1U	191		100.00	33.33	0.57	1.57
Average				74.46	48.71	0.55	3.51

T0570	3NO3	258	Metal, Non-metal	50.00	44.44	0.45	3.10
T0607	3PFE	471		57.14	32.00	0.41	2.97
T0615	3NQW	179		26.67	44.44	0.30	8.38
Average				44.60	40.29	0.39	4.82

T0515	3MT1	365	Non-metal	50.00	20.69	0.29	3.29
T0516	3NO6	229		30.77	50.00	0.36	5.68
T0524	3MWX	325		53.85	77.78	0.64	4.00
T0526	3NRE	290		11.11	5.56	0.04	3.10
T0547	3NZP	611		58.82	26.32	0.37	2.78
T0565	3NPF	326		41.67	62.50	0.50	3.68
T0584	3NF2	352		23.08	25.00	0.21	3.69
T0591	3NRA	406		41.67	83.33	0.58	2.96
T0597	3NIE	429		31.58	37.50	0.32	4.43
T0599	3OS6	399		7.69	11.11	0.07	3.26
T0604	3NLC	549		9.09	16.67	0.08	6.01
T0609	3OS7	340		70.00	70.00	0.69	2.94
T0613	3OBI	287		26.67	44.44	0.32	5.23
T0622	3NKL	138		20.00	37.50	0.21	10.87
T0632	3NWZ	168		18.75	37.50	0.21	9.52
T0636	3P1T	336		23.53	66.67	0.38	5.06
T0641	3NYI	296		11.11	66.67	0.26	6.08
Average				31.14	43.48	0.32	4.86

### Prediction results on CASP8

We also apply our method to evaluate on the CASP8 data set by training on the CASP9 data set. In the same way, we list the prediction performance on different sliding windows for encoding input vectors and obtain the average performance over those sliding windows. Results also show that the combination of different sliding windows yields better performance than any individual sliding window in a robust way, for there is no rule to determine the sliding window size in different data sets. In this work the best one is window 7 in CASP9 while window 5 and 27 in CASP8 (see Table 4). In Table 4 the combination technique yields an MCC of 0.44 while the best sliding window technique achieves an MCC of 0.43.

**Table 4:** Prediction performance on different sliding windows for encoding input vectors on the CASP8 data set.

	5	7	9	11	17	27	37	47	57	All
Sen(%)	48.00	51.06	63.30	51.43	53.74	45.27	49.24	58.53	51.25	52.11
Spe(%)	96.86	96.27	93.15	96.10	95.02	97.93	96.35	92.89	95.52	97.11
Acc(%)	93.93	93.46	90.97	93.31	92.37	94.65	93.41	90.59	92.73	94.19
MCC	0.42	*0.43*	0.42	0.42	0.39	0.43	0.39	0.37	0.38	*0.44*
Prec(%)	48.95	48.80	39.94	46.42	38.04	53.58	43.04	33.94	39.75	50.49
F1(%)	40.97	42.02	41.17	41.00	38.66	41.16	38.97	37.17	38.54	43.02

The ensemble of all sliding windows is shown at the last column of the table.

The italic numbers denote the best performance among different sliding windows on the measure of MCC, while the italic number in the last column is for the combination of all sliding windows.

Similar results of prediction performance on CASP8 data set can be shown in Table 5 comparing to that on the CASP9 data set. The average performance by MCC on those targets bound to metal ligands is better than that bound to non-metal ligands, where the former achieves an average MCC of 0.53 and the latter achieves an MCC of 0.32 only. In addition, although targets with metal ligands contain less number of binding sites than those with non-metal ligands, our method can identify them more accurately, except for two targets, T0410 and T0487.

**Table 5:** Performance and information on each of the CASP8 targets.

Target	Target PDB id	Number of residues	Ligand	Chemical Class	Sen (%)	Prec (%)	MCC	Ratio(%)^§^
T0391	3d89	157	FES	Metal	44.44	80.00	0.58	5.73
T0406	3di5	167	NI		100.00	100.00	1.00	1.80
T0407	3e38	363	ZN ZN ZN		88.89	66.67	0.76	2.48
T0410	3d3l	541	FE		12.50	6.90	0.06	2.96
T0425	3czx	181	ZN		33.33	100.00	0.57	4.97
T0426	3da2	283	ZN		33.33	18.75	0.22	3.18
T0440	3dcp	275	FE ZN FE		100.00	42.86	0.64	3.27
T0444	2vux	326	FE		75.00	8.82	0.24	1.23
T0453	3ded	91	CA CA CA		25.00	33.33	0.26	4.40
T0457	3dev	320	MG		100.00	19.05	0.43	1.25
T0461	3dh1	189	ZN		100.00	27.27	0.51	1.59
T0470	3djb	223	MG		100.00	26.67	0.50	1.79
T0476	2k5c	108	ZN		100.00	100.00	1.00	3.70
T0478	3d19	283	MG FE		71.43	100.00	0.84	2.47
T0480	2k4x	55	ZN		75.00	75.00	0.73	7.27
T0487	3f73	685	MG		25.00	5.00	0.10	0.58
Average					61.50	50.65		2.79

T0394	3dcy	275	PO4	Non-metal	33.33	57.14	0.42	4.36
T0396	396	105	FAD		13.04	75.00	0.26	21.90
T0422	3d8b	357	ADP		41.18	38.89	0.37	4.76
T0430	3dlz	357	AMP		14.29	37.50	0.20	5.88
T0431	3dax	491	HEM		10.53	18.18	0.11	3.87
T0450	3da1	561	FAD		41.03	27.59	0.28	6.95
T0477	3dkp	242	ADP MG		60.00	50.00	0.53	4.13
T0483	3dls	335	ADP MG		39.13	42.86	0.37	6.87
T0485	3dlc	218	SAM		5.26	100.00	0.22	8.72
T0490	3dme	369	FAD		29.41	35.71	0.26	9.21
T0508	3dou	197	SAM		36.84	70.00	0.47	9.64
Average					29.46	50.26		7.84

^§^It stands for the ratio of the number of ligand sites to the total number of residues of the protein chain.

### Comparison with other binding site prediction methods

**Figure 2:**
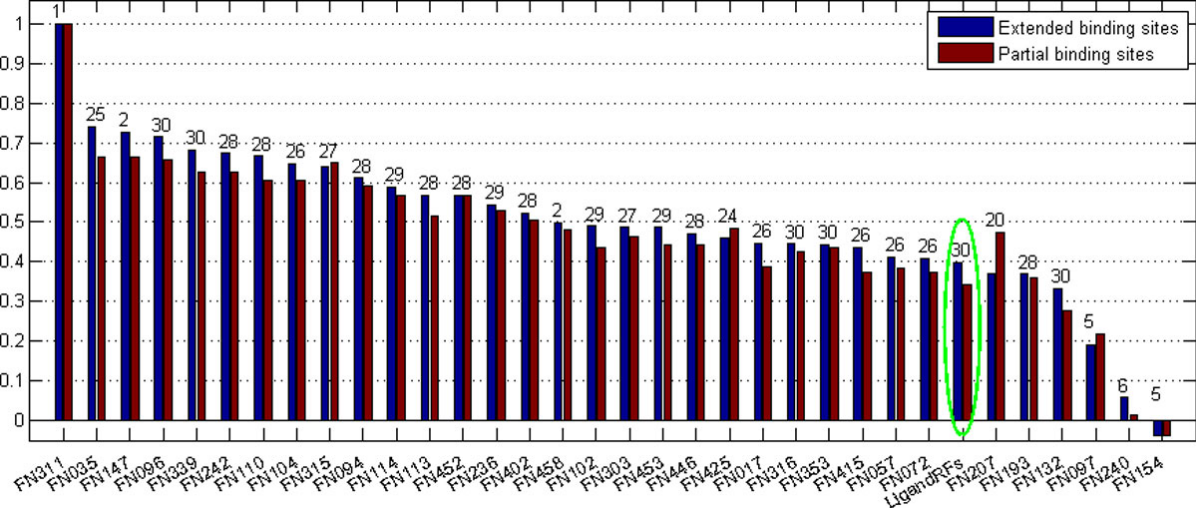
**Performance comparison of different methods on the measure of MCC for the partial binding site group and the all binding site group**. The number above each bar denotes the number of protein targets the method was tested on, and the green eclipse shows our predictor. Here we list all predictors in CASP9 including those tested on even one protein target.

**Figure 3:**
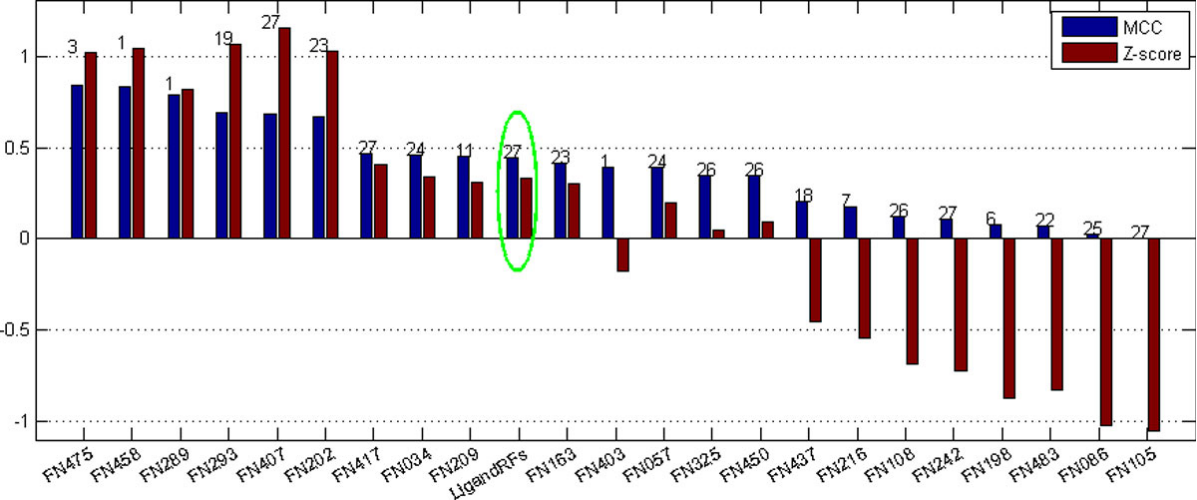
**Performance comparison of different methods on the measures of MCC and Z-score**. The number above each bar denotes the number of protein targets the method was tested on, and the green eclipse shows our predictor. Here we list all predictors in CASP8 including those tested on even one protein target.

Previous experiments showed that template-based prediction methods will perform much better than de novo methods in the context [1], but our method provides a comparative prediction on protein ligand binding sites, especially for the CASP8 data set. Figures 2 and 3 illustrate prediction comparison on CASP9 and CASP8 data sets, respectively. Although our method performs worse than most of template-based methods on the CASP9 data set, it performs better than many methods on the CASP8 data set.

It is difficult to compare our model with other similar methods for there are seldom methods of predicting ligand binding sites by using only sequence information. Most of ligand binding site prediction methods applied structural information of homologous proteins for the prediction. In CASP9, FN0193 is a predictor using SVM to identify protein binding sites. It basically used sequence profile information, the results from the disorder prediction models as well as secondary structure prediction models as additional features for the ligand-binding prediction models. Another work using sequence information was FN0132, which combined sequence information and homology-based transfer to identify protein binding sites. Our method yields an MCC of 0.40, which outperforms the two methods. Other two sequence-based methods in Table 6 performed even worse, only achieving an MCC of 0.19 for FN097 and 0.06 for FN240 in CASP9. Moreover, the random predictor is also implemented here and ran 100 times. The average performance is appended at the last row of Table 6. Results show that our method outperforms the random predictor by 36 times of the MCC score and 6 times of the F1 score.

**Table 6:** Performance comparison of the six methods on the CASP9 data set.

Method	Type	# of targets	Sen (%)	MCC	Prec (%)	F1 (%)
LigandRFs	Random Forest	30	42.07	0.40	47.85	38.93
FN0193	SVM	28	42.95	0.37	39.20	37.18
FN0132	SVM (LIBRUS)	30	57.37	0.33	25.46	33.55
FN097	Hydrophobicity-probability	5	15.28	0.19	28.57	19.00
FN240	Network centrality	6	14.91	0.06	8.53	10.11
Random Pre	dictor	30	0.10	0.01	0.05	0.06

The third column denotes how many targets in CASP9 are tested in the evaluation of each method. The details for FN0193, FN1032, FN097, and FN240 can be referred to [17].

### Case studies

**Figure 4:**
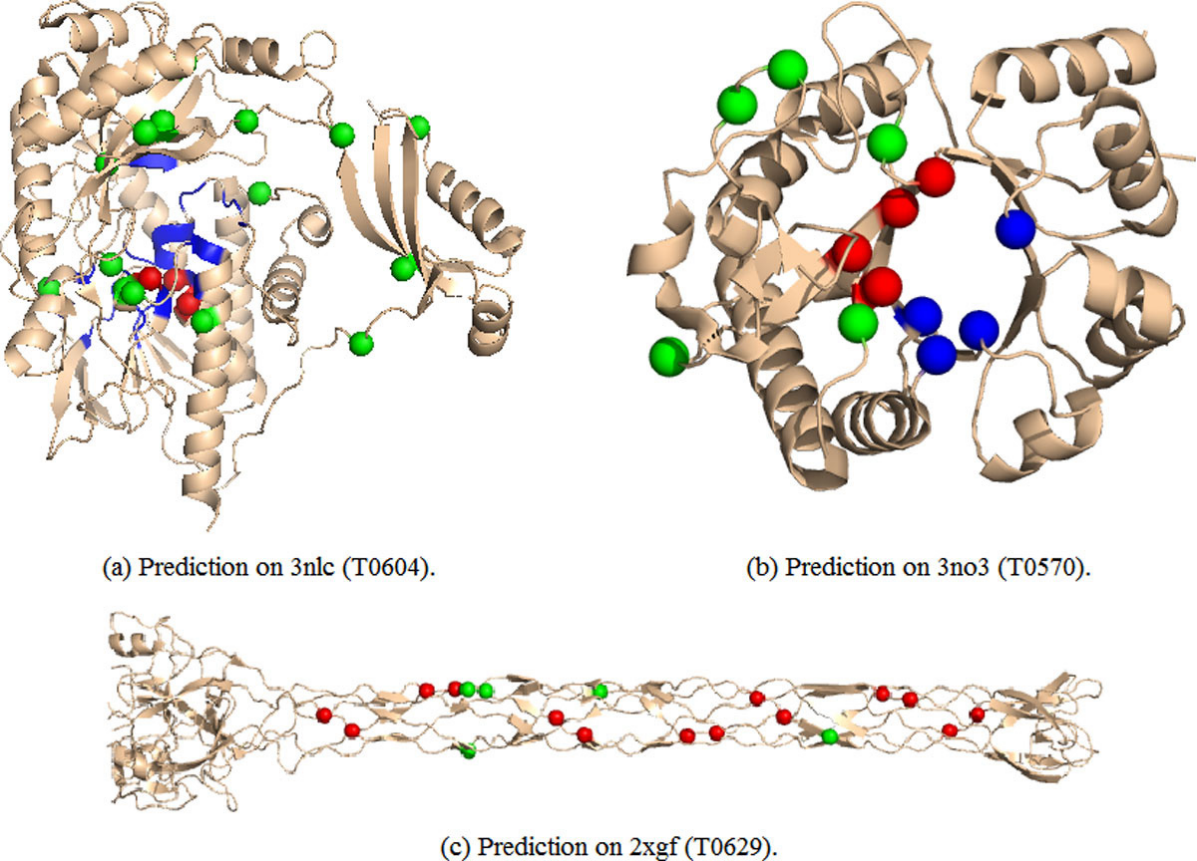
**Illustration of protein binding site prediction for Targets T0570 (a), T0604 (b), and T0629 (c)**. (a) Prediction on 3nlc (T0604); (b) prediction on 3no3 (T0570); (c) prediction on 2xgf (T0629). Here the correctly predicted binding site residues are colored in red, the wrongly predicted binding sites in green, and the wrongly predicted non-binding sites in blue.

Two targets in CASP9 were free modeling (FM) targets. The first one was T0604 (PDB: 3nlc), which is a putative FAD-dependent oxidoreductase with a bound FAD molecule. Experiments from CASP9 showed that the target was the most difficult one in the FN prediction in CASP9, with a maximum MCC of 0.56 and an average score of 0.29. Our sequence-based predictor yields an MCC of 0.08. The prediction of our method on T0604 is shown in Figure 4(a). Although the prediction is not good, our method can identify ligand binding sites partially. In addition, some wrongly predicted binding sites are around those true binding sites.

Another FM target was T0629 (PDB: 2xgf). It is formed by three chains and binds seven FE ions. Each FE ion is complexed by six histidine residues, where each two histidine residues is from one chain. For the same structures of the three chains, only prediction on chain A is illustrated in Figure 4(c). Experiments in CASP9 showed that all predictors in CASP9 can correctly identified a subset of the seven binding sites. Our method can cover all of the true binding sites, and only contain five wrongly predicted binding sites where two ones are close to true binding sites. Our method yields an MCC up to 0.85 for the target, which outperforms most of the methods in CASP9.

The last case is for the target T0570 (PDB: 3no3), which binds GOL nonmetal ligand coordinated to the MG metal ion. In target T0570, residues His30, Glu59, Glu123, Ile156, Phe158, Leu178 and Trp222 are bound to the GOL nonmetal ligand, while residues Glu59, Asp61 and Glu123 are bound to the MG metal ion. Our method can identify four binding site residues: His30, Glu59, Asp61, Glu123, some of which (His30, Glu59 and Glu123) bound to the GOL ligand and some (Glu59, Asp61 and Glu123) bound to the MG ion. Although our predictor performs worse than some structure-based methods in CASP9, it can cover half of the true binding sites and yield an MCC of 0.45 for the target T0570.

## Discussion

Experimental results showed that structure-based predictors yielded worse predictions on targets without local homologues [1,12]. Target T0604 is a typical case. It yielded a maximum MCC score of 0.56 for the best prediction, and an average score of 0.29. Actually since the target has only remote homologues, its sequence profile is much sparser than other targets. The final encoding vectors for expressing the residues of the target cannot reflect the evolutionary context of binding sites. The following Table 7 shows part of sequence profile for target T0604, where most of the elements are zero. Therefore, other features such as secondary structure information as well as other physico-chemical characteristics of residues should be addressed and incorporated as input features, and thus might improve the prediction based on sequence features.

**Table 7:** Part of the sequence profile for target T0604.

...	...	...	...	...	...	...	...	...	...	...	...	...	...	...	...	...	...	...	...	...
510	0.0	0.0	0.0	0.0	0.0	1.0	0.0	0.0	0.0	0.0	0.0	0.0	0.0	0.0	0.0	0.0	0.0	0.0	0.0	0.0
511	0.0	0.0	0.0	0.0	0.59	0.0	0.0	0.0	0.0	0.41	0.0	0.0	0.0	0.0	0.0	0.0	0.0	0.0	0.0	0.0
512	0.0	0.0	0.0	0.0	0.23	0.0	0.0	0.0	0.0	0.0	0.0	0.0	0.0	0.0	0.0	0.0	0.0	0.0	0.0	0.77
**513**	0.0	0.0	0.0	0.0	0.0	0.0	0.0	0.0	0.0	0.0	0.0	0.0	1.0	0.0	0.0	0.0	0.0	0.0	0.0	0.0
**514**	0.96	0.0	0.0	0.0	0.0	0.01	0.0	0.0	0.0	0.0	0.0	0.0	0.0	0.0	0.0	0.03	0.0	0.0	0.0	0.0
515	0.0	0.0	0.0	0.0	0.0	1.0	0.0	0.0	0.0	0.0	0.0	0.0	0.0	0.0	0.0	0.0	0.0	0.0	0.0	0.0
516	0.0	0.0	0.0	1.0	0.0	0.0	0.0	0.0	0.0	0.0	0.0	0.0	0.0	0.0	0.0	0.0	0.0	0.0	0.0	0.0
**517**	0.0	0.0	0.0	0.0	0.0	1.0	0.0	0.0	0.0	0.0	0.0	0.0	0.0	0.0	0.0	0.0	0.0	0.0	0.0	0.0
518	1.0	0.0	0.0	0.0	0.0	0.0	0.0	0.0	0.0	0.0	0.0	0.0	0.0	0.0	0.0	0.0	0.0	0.0	0.0	0.0
519	0.0	0.0	0.0	0.0	0.0	1.0	0.0	0.0	0.0	0.0	0.0	0.0	0.0	0.0	0.0	0.0	0.0	0.0	0.0	0.0
520	0.0	0.0	0.0	0.0	0.0	0.0	0.0	0.0	0.0	0.0	0.0	0.0	0.0	0.0	0.0	0.0	0.0	0.0	0.0	1.0
...	...	...	...	...	...	...	...	...	...	...	...	...	...	...	...	...	...	...	...	...

The first column is residue number in the target sequence, while columns 2 to 21 are acid/amide forms in proportion to their blasted database frequencies.

There is no evidence to show that binding sites to metal ions are easier to be identified than that to nonmetal ligands for those structure-based methods [12,20], although non-metal ligands are larger and more residues will bind non-metal ligands than metal ligands. However, in this work our sequence-based method yields good prediction on targets bound to metal ions, and achieves an MCC of 0.55 for the CASP9 data set and 0.53 for the CASP8 data set; while predictions on targets bound to non-metal ligands are much worse. It might be that residues bound to metal ligands are more conserved in evolutionary context than that bound to non-metal ligands, and thus the former can be identified more accurate.

Moreover, prediction performance may be changed with different sliding window sizes. To reduce the effects, we took Eq. 1 to address the issue. Results on both the CASP8 and the CASP9 data sets show the success and are shown in Tables 4 and 2. The combination of sliding windows performs better than all of the individual ones, it achieves an average MCC of 0.40 (0.37 for the best individual one) for the CASP9 data set and an average MCC of 0.44 (0.43 for the best individual one) for the CASP8 data set. Therefore the selection of the sliding window in different situations can be avoided.

## Conclusions

This paper proposes an ensemble of RF classifiers with only sequence information to predict ligand binding sites. In order to balance the ligand site data set, several data sets are constructed and composed of the binding site subset (positive subset) and one of the non-binding site subsets (negative subsets), each of the negative subsets is independent to the others. Then each data set is inputted into a RF classifier. The ensemble of these RF classifiers can yield good prediction on ligand-binding sites. The encoding schema integrating those properties and evolutionary information of amino acids is important to obtain the evolutionary context of ligand binding site residues and thus, the method yields good performance on predicting ligand binding sites. Although structure-based methods still outperform sequence-based methods, our method provides a potentially alternative solution to the binding site prediction problem, especially when similar structure information of the query is not available.

## Methods

### Data sets

We took the targets in the CASP9 competition as our ligand-binding site data set. As stated in CASP9, there were 30 targets with bound ligands, out of which 10 were found in complex with metal ions (Ca, Fe, Mg, Mn, Na, and Zn), 17 were in complex with non-metal ligands (ANP, BES, COA, CSA, DST, EDO, FAD, GAL, GAR, GLA, GOL, GPX, HSA, IMD, IPR, ISC, LLP, LYS, NAD, NHS, PEG, PF1, PLP, SO4, STE, TLA), and three were in complex with hybrid ligands. Moreover, ligand-binding sites in most of the targets were located within a monomer. However, in six targets, the ligand was bound in the interface between multiple chains [1]. The assignments of ligand-binding sites were carefully checked and only the targets with unambiguous assignment were retained.

In addition, we took another data set in the CASP8 meeting to validate our method. In CASP8, there were 27 targets bound to 37 ligands. Of the ligands, there are 26 metals in 18 targets (Mg, Zn, Fe, FeS, Ni, and Ca), nine nucleotides (ADP, AMP) or derivates (FAD, SAM), one metabolite (PO4) and the last target bound a heme moiety [20].

### Binding site definition

**Figure 5:**
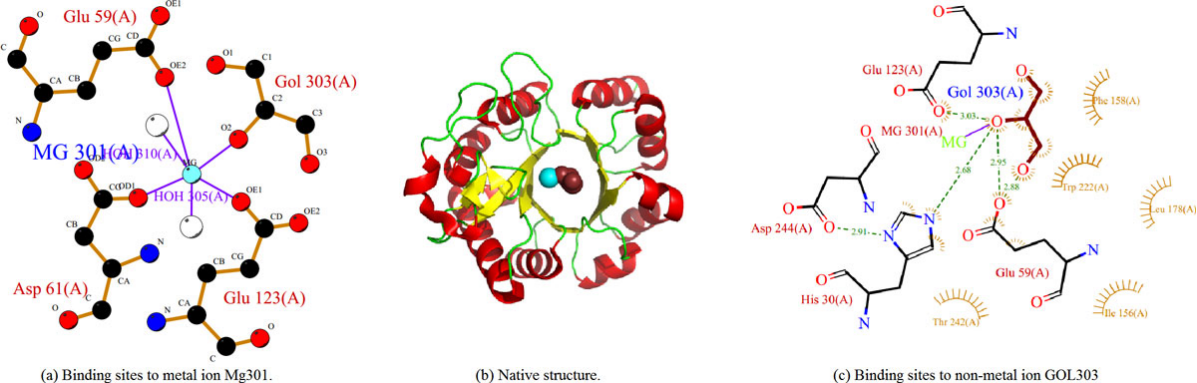
**LIGPLOT 38 of binding site residues to ligands for PDB ID, 3NO3**. (a) the binding site residues to metal ion Mg301 (colored in cyan); (b) the structure of protein 3NO3 (GOL303 is colored in ruby and Mg301 in cyan); (c) The binding sites to non-metal ion GOL303 (colored in ruby). In CASP9 experiment, only His30, Glu59, Asp61, Glu123, Ile156, Phe158, Leu178 and Trp222 are deemed as ligand binding site residues.

For each protein, all residues, at least one heavy atom within a given distance from any heavy atom of the ligand, were defined as ligand-binding site residues. In fact, the definition of the distance cutoff was different in literature. Kauffman and Karypis collected ligand-binding residues having at least one heavy atom within 5 Å of a ligand [18]. While in CASP9, the distance cutoff was defined as the sum of the van der Waals radii of the involved atoms plus a tolerance of 0.5 Å. Different distance cutoff leads to different ligand-binding site data set, i.e., about 9% of residues are the ligand-binding residues for the former definition, while only 3.9% for the latter. For the CASP9 definition, there are in total 355 ligand-binding residues and 8718 non-ligand binding residues of the 30 proteins. For the CASP8, there are 335 ligand-binding residues in a total of 7718 residues of the 27 protein targets. Figure 5 illustrates the binding site residues to ligands for protein PDB: 3NO3, where two ions, metal ion Mg and non-metal ion GOL, are bound to the protein.

### Feature vector representation of a residue

In the AAindex1 database [21], there are 544 amino acid properties. Many of these properties are highly correlated. We thus extracted relatively irrelevant properties with a correlation coefficient (CC) of 0.5. For each of the 544 properties, the CC to all the other properties was calculated and the number of related properties was counted. The 544 properties were thus ranked according to their numbers of related properties. From the top property, we removed from the list all the properties that were related to it. This was repeatedly done until no related pair existed in the list, which resulted in 34 properties.

For a residue *i *in a protein chain, the association among the neighboring residues is considered in this work. A sliding window that contains seven residues centered at the residue *i *is used to encode the features. An encoding schema integrating amino acid properties and sequence profile is used to represent the residue. The sequence profile for one residue created by PSI-Blast with default parameters [22] is then multiplied by each amino acid property. For instance, the profile *SP^k^, k *= 1,..., 7 for residue *k *in the seven residue window and one amino acid property scale, *AAP_j_*, are both vectors with 1 × 20 dimensions. Thereafter, $MSK_{j}^{k}=SP^{k}\times AAP_{j}$ for residue *k *represents the multiplication of the corresponding sequence profile by the scale, where × represents the element-wise product. In our previous work, we found out that the standard deviation of $MSK_{j}^{k}$ reflected the evolutionary variance of the residue *k *along with the amino acid property *AAP_j _*[23-25].

For the residue *i *and the amino acid property, *AAP_j_*, therefore, it is represented by a 1 × 7 vector *V_ij _*for the case of sliding window with 7 residues. For all of the 34 amino acid properties, the residue *i *is represented by a 1 × (7 * 34) = 1 × 238 vector; the corresponding target value *T_i _*is 1 or 0, denoting whether the residue is a ligand-binding residue or not. Our goal is to learn the relationship between the input vectors *V *and the corresponding target array *T*.

### Combine different sliding windows

In fact, each sliding window for encoding the input vector of each residue may cause deviation in investigating the relationship between the residue property profile and the ligand binding site. We use a combination technique to reduce the effects of sliding window selection. Suppose there are *N *predictions, *Pred_n_, n *= 1, . . ., *N*, resulted from *N *sliding windows, a new prediction can be obtained by

(1) $$Pred_{comb}=\bar{Pred}-\sqrt{\frac{1}{N}\overset{N}{\underset{n=1}{\sum }}{\left(Pred_{n}-\bar{Pred}\right)}^{2}},where\;\bar{Pred}=\frac{1}{N}\overset{N}{\underset{n=1}{\sum }}\left(Pred_{n}\right).$$

The first term in the right part of Eq. 1 is the mean of predictions resulted from *N *sliding windows and the second one shows the standard deviation of them.

### Ensemble of random forest classifiers

Machine learning techniques have played very important roles in various protein-related problems, such as B-factors prediction [26], domain identification [27], function annotation [28], membrane protein type prediction [29], and protein retrieval [30]. Here we propose to use the random forest model for the binding site prediction. A random forest [31] consists of an ensemble of simple tree predictors, each of which depends on a set of random features selected independently. It is capable of producing a response when presented by a set of predictor values. Therefore, the generalization error of a random forest depends not only on the individual trees but also significantly on the correlation between them. For the ligand-binding site prediction problem, the ensemble of simple trees votes for the most popular ligand-binding site class. Previous results showed that using consensus ideas can make significant improvement in prediction accuracy [32-36].

Given a set of training data *D_N _*= {(*X_i_, Y_i_*)}, *i *= 1,..., *N*, let the number of training instances be *N*, the number of features in the classifier be *J*, and the number of trees to build be *K*. For each tree, a number of *j *features are considered to determine the decision of the tree, where *j *should be much less than *J *and set as 1 *≤ j ≤ int(log(J*) + 1) by default. For the *k − th *tree, a random vector *ϑ_k _*is generated, which is independent and with the same distribution of the previous ones, *ϑ*_1_,..., *ϑ_k−1_*. The *k − th *tree generated from the training set and *ϑ_k _*results in a classifier *CF_k _(x; ϑ_k_*), where *k *= 1,..., *K *and *x *is a training instance.

After all of the trees are generated, they vote for the most popular class and thus the prediction of the whole forest is,

(2) $$F\left(X\right)=majority\;vote{\left\{CF_{k}\left(X\right)\right\}}_{k=1}^{K},$$

where *X *is a query instance.

Since the binding site data set is highly imbalanced, i.e., only 3.9% of all the instances are positive samples, balancing the positive (binding site class) and the negative (non-binding site class) data is necessary to avoid the overfitting of classifiers. Since protein chains contain different ratios of binding sites to non-binding sites, 15 data sets are thus formed, $D_{N}^{n}$, *n *= 1,..., 15, each of which involves roughly the same number of the positive and negative samples. That is, the 15 data sets share the same positive samples, but have disjoint negative samples. A random forest classifier is trained for each of the 15 data sets. The final prediction is the majority voting of the 15 random forests.

### Evaluation criteria

In this work we adopted four evaluation measures to evaluate the performance of our method, i.e., sensitivity (Sen), precision (Prec), F-measure (F1), specificity (Spe), accuracy (ACC), and Matthews correlation coefficient (MCC) [23,37]. They are defined as follows:

(3) $$\begin{array}{c} Sen=\frac{TP}{TP+FN},Prec=\frac{TP}{TP+FP},F1=2\times \frac{Prec\times Sen}{Prec+Sen},\\ Sen=\frac{TN}{FP+TN},Acc=\frac{TN+TP}{TN+FP+FN+TP},\\ MCC=\frac{TP\times TN-FP\times FN}{\sqrt{\left(TP+FN\right)\left(TP+FP\right)\left(TN+FP\right)\left(TN+FN\right)}}, \end{array}$$

where TP (True Positive) is the number of correctly predicted binding sites; FP (False Positive) is the number non-binding sites that are predicted to be binding sites; TN (True Negative) is the number of correctly predicted non-binding sites; and FN (False Negative) is the number of binding sites that are predicted to be non-binding sites.

Besides, Z score is also used to evaluate the performance of our method. It can be used to reduce the effects of target difficulty on the ranking. The Z score of the predictor *P *for a given target *T *is shows as:

(4) $$Z_{P,T}=\frac{MCC_{P,T}-\bar{MCC_{T}}}{\sigma_{T}},$$

where *MCC_P,T _*is the raw *MCC *score for the target *T *given by the predictor *P*, $\bar{MCC_{T}}$ is the mean *MCC *score for the target *T*, and *σ_T _*is the standard deviation of *MCC *scores for the target *T*. The final Z score for the predictor *P *is the mean of Z scores over all targets.

## Competing interests

The authors declare that they have no competing interests.

## Authors' contributions

PC and XG conceived the study; PC participated in the experimental design; PC, JH and XG carried it out and drafted the manuscript. All authors revised the manuscript critically.
